# A net-shaped multicellular formation facilitates the maturation of hPSC-derived cardiomyocytes through mechanical and electrophysiological stimuli

**DOI:** 10.18632/aging.101411

**Published:** 2018-04-14

**Authors:** Taoyan Liu, Chengwu Huang, Hongxia Li, Fujian Wu, Jianwen Luo, Wenjing Lu, Feng Lan

**Affiliations:** 1 Beijing Laboratory for Cardiovascular Precision Medicine, The Key Laboratory of Remodeling-Related Cardiovascular Disease, Ministry of Education, Beijing Collaborative Innovation Center for Cardiovascular Disorders, Anzhen Hospital, Capital Medical University, Beijing 100029, China; 2 Beijing Institute of Heart, Lung and Blood Vessel Diseases, Beijing 100029, China,; 3 Department of Biomedical Engineering, School of Medicine, Tsinghua University, Beijing 100084, China; 4 Center for Biomedical Imaging Research, Tsinghua University, Beijing100084, China

**Keywords:** hiPSC, cardiac differentiation, cardiomyocyte clusters, maturation, motion analysis, ultrastructure

## Abstract

The use of human-induced pluripotent stem cell-derived cardiomyocytes (hiPSC-CMs) is limited in drug discovery and cardiac disease mechanism studies due to cell immaturity. Although many approaches have been reported to improve the maturation of hiPSC-CMs, the elucidation of the process of maturation is crucial. We applied a small-molecule-based differentiation method to generate cardiomyocytes (CMs) with multiple aggregation forms. The motion analysis revealed significant physical differences in the differently shaped CMs, and the net-shaped CMs had larger motion amplitudes and faster velocities than the sheet-shaped CMs. The net-shaped CMs displayed accelerated maturation at the transcriptional level and were more similar to CMs with a prolonged culture time (30 days) than to sheet-d15. Ion channel genes and gap junction proteins were up-regulated in net-shaped CMs, indicating that robust contraction was coupled with enhanced ion channel and connexin expression. The net-shaped CMs also displayed improved myofibril ultrastructure under transmission electron microscopy. In conclusion, different multicellular hPSC-CM structures, such as the net-shaped pattern, are formed using the conditioned induction method, providing a useful tool to improve cardiac maturation.

## Introduction

Cardiovascular disease is currently the leading cause of death worldwide. Despite an increased effort toward basic research and drug discovery, many cardiovascular diseases still have no curative treatments. As a type of terminally differentiated cells, the application of cardiomyocytes (CMs) is hindered by the difficulties in obtaining human cardiac tissue and the inability to propagate heart samples in culture. Therefore, most existing research has utilized animal models to mimic human heart disease. However, critical physiological and biochemical differences exist between the animal and human heart, especially for CMs. Fortunately, human-induced pluripotent stem cell (hiPSC) technology plays a vital role in the advancement of cardiovascular research and medicine and includes refined protocols for hiPSC reprogramming and cardiac differentiation that enable the derivation of human CMs with patient-specific phenotypes. This technology shows great potential in regenerative therapy and mechanistic investigations of cardiac diseases [1]. Previously, several hereditary cardiomyopathies were modeled *in vitro* using hiPSC technology, including hypertrophic cardiomyopathy (HCM) [2], dilated cardiomyopathy (DCM), long QT syndrome (LQT) [3] and LEOPARD syndrome . However, these *in vitro*-derived hiPSC-CMs are generally immature, with features resembling fetal CMs rather than adult myocardial tissue [4,5].

Unlike adult CMs, cultured hiPSC-CMs exhibit less organized sarcomeres and fewer mitochondria [6]. They also express much lower levels of genes that encode contractile proteins, comparable to fetal CMs, according to transcriptional profiling. Regarding the electrophysiological properties, hiPSC-CMs present immature action potentials [7], poorly developed SR and altered Ca^2+^ handling properties [8-10] at early stages of differentiation [8,11] as well as the absence of transverse tubules, automaticity, and a preference for glucose metabolism over fatty acid metabolism [12], which are consistent with immature phenotypes. hiPSC-CMs are also limited in their ability to detect the mechanism of cardiac diseases. Lan et al. [2] generated disease-specific iPSC-CMs from patients carrying a hereditary HCM missense mutation in the *MYH7* gene, but the diseased iPSC-CMs displayed the HCM phenotype after prolonged culture (40 days). Hinson et al. [13] used engineered biomimetic culture systems to assess the mechanism of DCM caused by *TTN* mutation. Although various pro-maturation methods (prolonged culture time [14], mechanical stretch [15], electrical stimulation [4,11,16], modulation of substrate stiffness [17,18], cell aggregation patterning [19,20], chemical stimulation [21,22], and the incorporation of CMs into 3D tissue constructs [23]) have all been attempted with varying degrees of success, researchers have not clearly determined whether the physical formation of the multicellular structure affects the maturation of hiPSC-CMs.

Therefore, the elucidation of the maturation status, which has a fundamental effect on the physical and biochemical properties of hiPSC-CMs, is crucial. In this study, we hypothesized that different forms of CM aggregates would have different maturation statuses.

## RESULTS

### The starting density of stem cells produces different CM clusters with multiple forms

**Figure 1:**
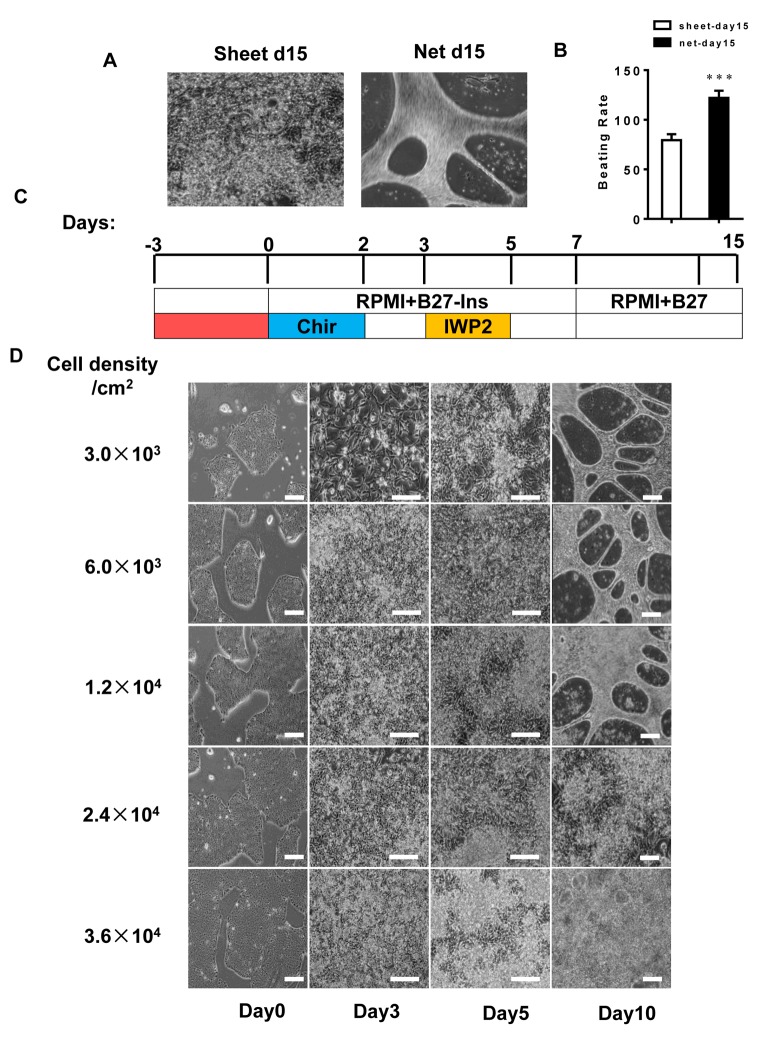
**The hiPSC seeding density is a determining factor in differentiating the two forms of cardiomyocyte clusters.** (**A** and **B**) The beating rates of two forms of hiPSC-CMs; left panel: sheet-shaped CMs; right panel: net-shaped CMs. The movies are shown in the supplemental movies. (**C**) Time course of pluripotent growth and subsequent cardiac differentiation showing the medium and small molecules used on each day of differentiation. (**D**) Representative images of hiPSCs seeded at different densities on E8 and subsequently differentiated.

To generate CMs from pluripotent stem cells, we applied a small molecule-based differentiation method. Upon differentiation, we observed three typical forms of CM clusters: 1) isolated beating clusters at a lower efficiency and 2) net-shaped and 3) sheet-shaped CMs at higher efficiencies. When the efficiency/purity was greater than 90%, the derived CMs predominantly exhibited the latter forms. Spontaneous contraction could be observed from 7 days after induction, and the different shapes of CM clusters became distinguishable. The net-shaped cardiac cells beat vigorously, with a beating rate of up to 122 beats per min, and the sheet-shaped cardiac cells beat mildly at a lower rate of 80 beats per min (Figure 1A, B, Movie S1 and Movie S2).

To determine the factors involved in the derivation of net-shaped and sheet-shaped CMs, we changed the seeding densities and the number of days prior to cardiac induction. As shown in Figure 1C and D, a seeding density ranging between 3.0×10^3^ and 1.2×10^4^/cm^2^, which should dramatically impair the efficiency of CM production, led to 83.3-100% efficient net-shaped CM production. A higher density led to a less than 10% efficient net-shaped CM production. Under the defined condition of cardiac differentiation, these results are very well reproducible with a success rate of greater than 80% in more than 50 repeats. We performed flow cytometry using the cardiomyocyte marker TNNT2 to validate the efficiency of differentiating CMs with different shapes. The results showed a similar yield of TNNT2+ cells in the two shapes of CMs (Figure S1).

### Net-shaped CMs are more mature than sheet-shaped CMs

**Figure 2:**
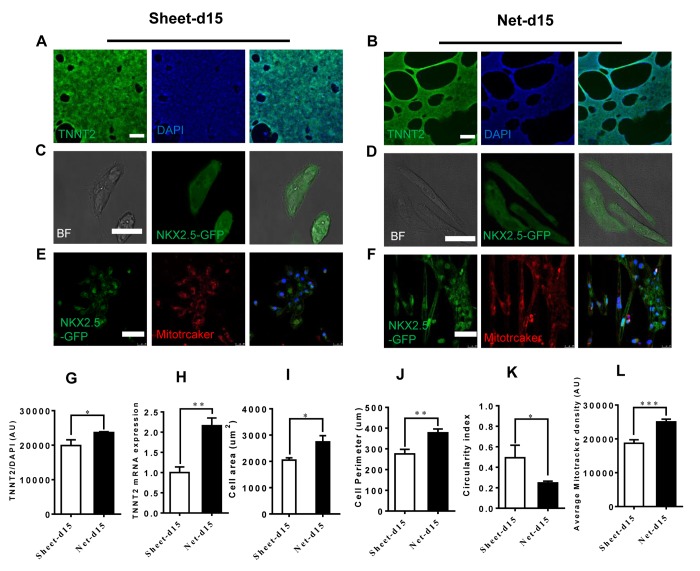
**Improved cardiomyocyte contractility in net-shaped hPSC-CMs. **(**A-F**) Immunostaining of sheet-shaped hiPSC-CMs 15 days after cardiac differentiation. (**A** and **B**) Images of cTnT (green) and DAPI (blue) staining. (**C** and **D**) NkX2.5-GFP and bright field images; (**E** and** F**) MitoTracker images (red) (**G**) cTnT fluorescence intensity; (**H**) TNNT2 mRNA expression. (**I, J, **and** K)** Cell area, cell perimeter, circularity index (n=51 in net-d15 and n=45 in sheet-d15). (**L**) Average MitoTracker density; All data were normalized to the sheet-day 15 group and are expressed as means±S.E.M. * Statistically significant differences between individual groups (n≥3; *P<0.05, **P<0.01, ***P<0.001). Scale bar: 50 μm.

During development, CMs undergo physiological hypertrophy characterized by increased cell size followed by changes in sarcomere structure, mitochondria content and organization and increased protein synthesis [6,11,24]. We performed immunostaining on both CM clusters after isolation using the CardioEasy CM dissociation enzyme to explore differences in maturity between the net-shaped and sheet-shaped CMs. Immunostaining revealed increased troponin expression in net-shaped CMs, which was confirmed by the quantitative mRNA expression analysis (Figure 2A, B, G, and H). After the clusters were dissociated into single cells, we analyzed the cellular area, cell perimeter and circularity index, and the net-shaped CMs displayed larger sizes and had higher aspect ratios (Figure 2C and I-K). As shown in Figure 2E and F, the mitochondria were more abundant in net-shaped CMs presented a better aligned distribution, particularly the perinuclear localization (Figure 2L). The results implied that the net-shaped CMs had more mature structural properties than the sheet-shaped CMs.

### Net-shaped CMs displayed accelerated maturation at the transcriptional level

**Figure 3:**
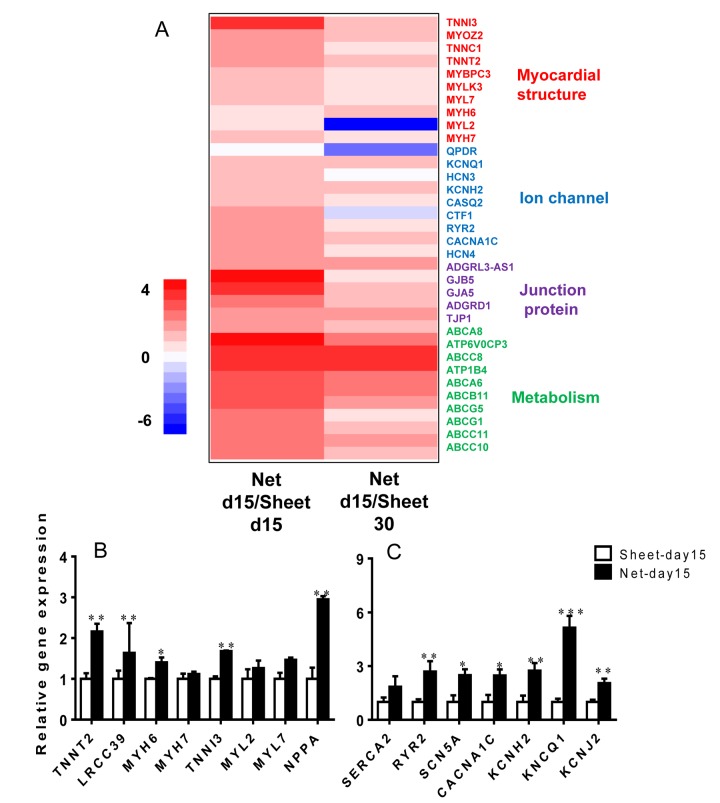
**The net-shaped CMs act more mature at the transcriptional level than the sheet-shaped CMs.** (**A**) NOISeq method of screening differentially expressed genes between net-shaped CMs and sheet-shaped CMs; some highly up-regulated genes are shown in a heat map. (**B**) Real-time quantitative polymerase chain reaction analyses of cardiac structure-related genes (*TNNT2*, *LRCC39*, *MYH6*, *MYH7*, *MYL2*, and *MYL7*) and cardiac function-related genes (*SEARA2A*, *RYR2*, *SCN5A*, *CACNA1C*, *KCNH2*, *KCNQ1*, and *KCNJ2*) to verify the mRNA levels in the sheet-shaped and net-shaped CMs. * Statistically significant differences between individual groups (n≥3; *P<0.05, **P<0.01, ***P<0.001).

To assess the overall maturation status of the net-shaped and sheet-shaped CMs, we used RNAseq to examine the global gene expression profiles of the net-shaped CMs, sheet-shaped CMs and the long-term cultured CMs (30 days). Compared to the sheet-shaped CMs, the expression of 482 genes was decreased and the expression of 348 genes was increased in the net-shaped CMs. Meanwhile, 492 down-regulated and 410 up-regulated genes were observed in the net-shaped CMs compared with the long-term cultured CMs. We then performed a clustering analysis of differentially expressed genes to elucidate the mechanism of maturation induced by multicellular formation. The net-d15 CMs expressed higher levels of genes encoding contractile proteins, ion channels, junction proteins and metabolism-related proteins than the sheet-d15 CMs. Moreover, the fold change in cardiac maturation-related gene expression decreased in net-d15/sheet-d30 compared with the net-d15/sheet-d15, indicating that the net-shaped CMs were more similar to long-term cultured CMs (30 days) than to sheet-d15 (Figure 3A). We also analyzed the cellular area, cell perimeter, circularity index of the sheet-d15, net-d15 and sheet-d30. As shown in Figure S2, net-shaped CMs displayed cell areas that were similar to sheet-d30, but they had larger cell perimeters and lower circularity indexes (Figure S2). Based on these results, the maturation of net-shaped CMs was accelerated.

### Ion channel genes were up-regulated in the net-shaped CMs to adapt to robust contraction

To confirm the expression levels of cardiac genes in the net-shaped and sheet-shaped CMs, we performed qRT-PCR on cells from both groups. As shown in Figure 3B and C, higher levels of the structural genes *TNNT2, TNNI3, MYH6, MYL2, MYL7*, and leucine-rich repeat-containing protein 39 (*LRRC39*) were observed in the net-shaped CMs than in the sheet-shaped CMs. Moreover, the expression levels of major cardiac ion channel proteins, such as sarco/endoplasmic reticulum Ca^2+^ ATPase (*SEARCA2*), ryanodine receptor 2 (*RYR2*), Na_v_1.5 (*SCN5A*), Ca_v_1.2 (*CACNA1C*), hERG (*KCNH2*), K_v_7.1 (*KCNQ1*) and K_ir_2.1 (*KCNJ2*), were all significantly increased in the net-shaped CMs compared with the sheet-shaped CMs. Thus, the net-shaped CMs exhibit a more mature function than the sheet-shaped CMs.

### Motion analysis revealed significant physical differences in CMs of different shapes

**Figure 4:**
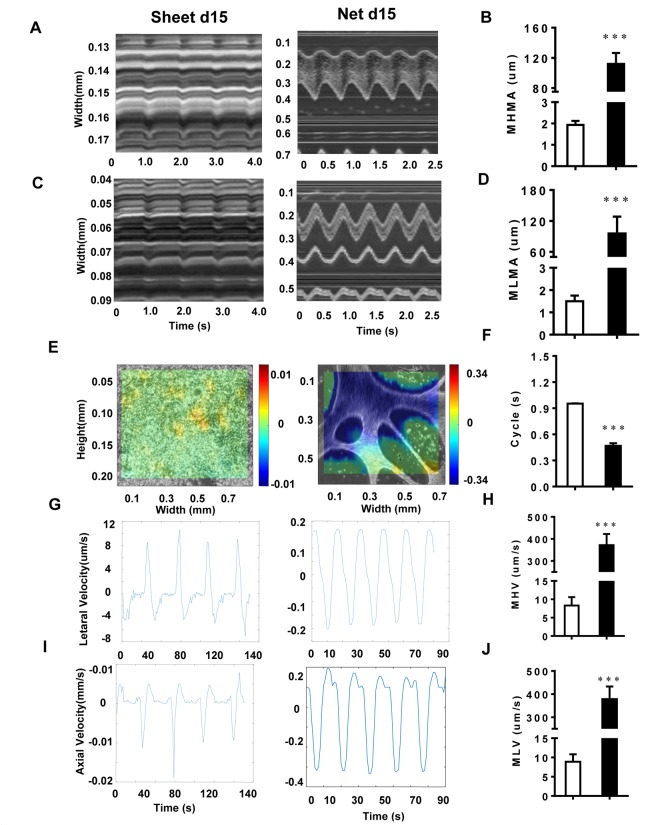
**The motion analysis reveals significant physical differences among CMs with different shapes.** (**A** and **B**) The MHMA of sheet-shaped CMs and net-shaped CMs; left panel: sheet-shaped CMs; right panel: net-shaped CMs. (**C** and **D**) The MLMA of sheet-shaped and net-shaped CMs; left panel: sheet-shaped CMs; right panel: net-shaped CMs. (**E**) The direction and velocity of two forms of hiPSC-CMs. Movement to the right is shown in red and to the left is shown in blue; deeper colors represent a greater velocity. (**F)** Movement cycles of the two forms of hiPSC-CMs. (**G** and **H**) The MHV of sheet-shaped and net-shaped CMs. (**I** and **J**) The MLV of sheet-shaped and net-shaped CMs; left panel: sheet-shaped CMs; right panel: net-shaped CMs. All data are expressed as means±S.E.M. * Statistically significant differences between individual groups (n≥3; *P<0.05, **P<0.01, ***P<0.001).

To further investigate the behavior of the two CM cluster shapes, we used a cross-correlation-based speckle tracking method to analyze the movement of the CMs. The results are shown in Figure 4. The maximum horizontal motion amplitude (MHMA) of the net-shaped CMs was 111.96±14.64 μm, and the maximum longitudinal motion amplitude (MLMA) was 95±32.8 μm. The MHMA and MLMA of the sheet-shaped CMs were 1.93±0.18 μm and 1.50±0.25 μm, respectively, which were significantly lower than the net-shaped CMs (Figure 4A-D). Moreover, the maximum velocity of the net-shaped CMs was significantly faster than that of the sheet-shaped CMs. The maximum horizontal velocity (MHV) of the net-shaped CMs was 372.00±50.17 μm/s, and the maximum longitudinal velocity (MLV) was 378.50±54.60 μm/s. The MHV and MLV of the sheet-shaped CMs were 8.30±2.31 μm/s and 8.90±1.92 μm/s, respectively (Figure 4E-J). Meanwhile, the motion cycle time of the net-shaped CMs (0.47±0.03 s) was significantly shorter than that of the sheet-shaped CMs (0.95±0.003 s) (Figure 4F).

### Net-shaped CMs displayed improved myofibril ultrastructure

**Figure 5:**
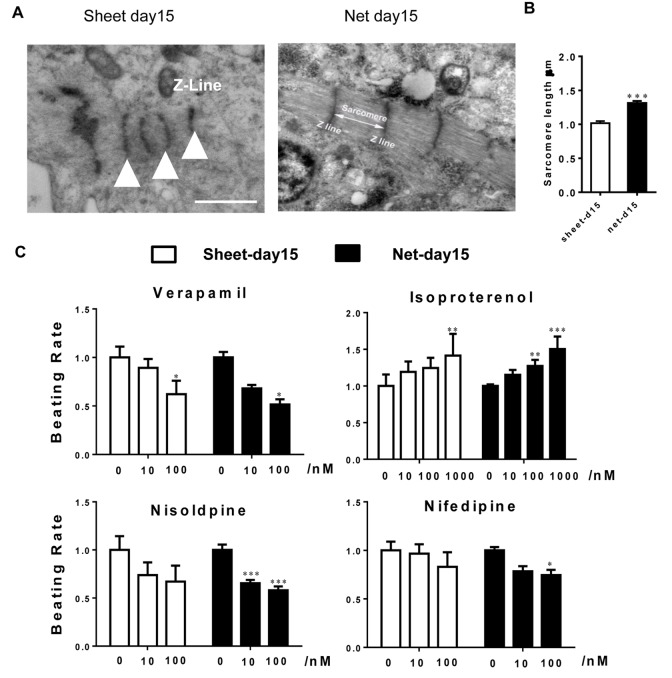
**Ultrastructural analyses and drug responses further confirm net-shaped CM maturation.** (**A**) Transmission electron microscopy images of human stem cell-derived CMs 15 days after cardiac differentiation. Scale bar: 1 μm; sarcomere: white triangle. (**B**) Pharmacological responses of hiPSC-CMs to cardiac-active compounds. Upper panel: nisoldipine and nifedipine; lower panel: verapamil and isoproterenol. All data were normalized to the sheet-day 15 group and are expressed as means±S.E.M. * Statistically significant differences between individual groups (n≥3; *P<0.05, **P<0.01, ***P<0.001).

When investigating the ultrastructure of the two types of CMs, we found that, at 15 days of differentiation, hiPSC-CMs contained myofibrils that lacked alignment or an organized sarcomeric pattern and were distributed diffusely in the cytoplasm in a disorganized manner. Scattered patterns of condensed Z-bodies were also observed. However, in some areas, a more developed pattern could be observed at the ultrastructural level, with sarcomeres in sheet-shaped CMs that were thin, disorganized and oriented in multiple directions. In contrast, the net-shaped CMs showed a remarkably well-developed striated ultrastructure with thick sarcomere subunits, as shown in Figure 5A and B.

### Net-shaped CMs are more sensitive to lower doses of pharmacological stimuli

To assess the pharmacological responses of the net-shaped and sheet-shaped CMs, we treated the cells with several typical compounds that regulate cardiac functions, including the beta-adrenergic receptor agonist isoproterenol, the K_v_11.1 and Ca_v_1.2 channel antagonist verapamil, and the L-type Ca^2+^ channel antagonists nifedipine and nisoldipine. As shown in Figure 5C, both the net-shaped CMs and the sheet-shaped CMs positively responded to isoproterenol and negatively responded to verapamil, nifedipine and nisoldipine. In addition, upon treatment with isoproterenol, nisoldipine and nifedipine, net-shaped CMs were more sensitive to lower doses of compounds, as indicated by the relative changes in the beating rate.

### Robust contraction was coupled with enhanced connexin expression

**Figure 6:**
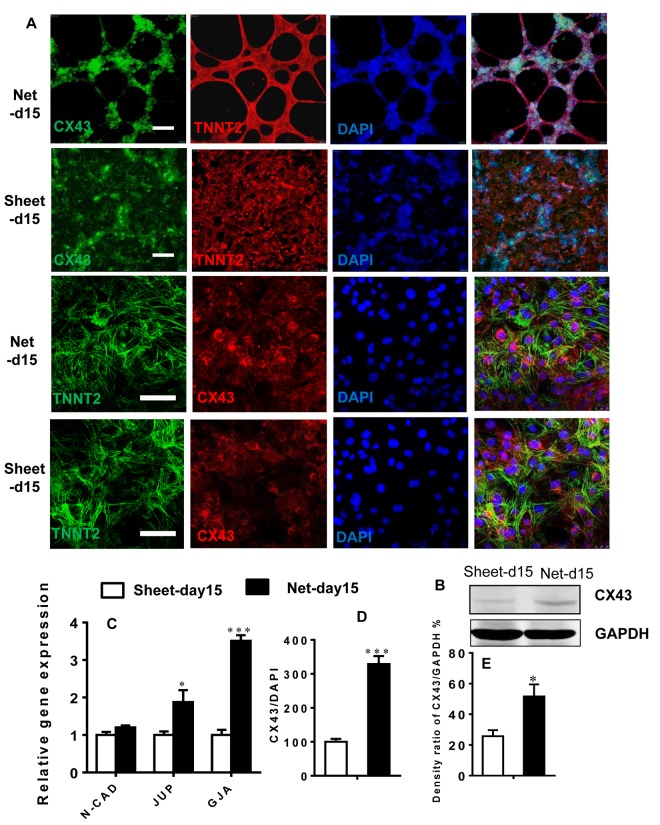
**Robust beating of net-shaped CMs enhances connexin expression.** (**A** and **D**) Immunostaining showing increased levels of connexin-43 (green) in net-shaped CMs. Troponin (red) and DAPI (blue) staining are also shown. (**C**) Quantitative PCR analysis of *N-CAD*, *JUP*, and *CX43* expression. (**B** and **E**) Up-regulation of the CX43 protein in net-shaped CMs. All data are expressed as means±S.E.M. (n≥3; ***P<0.001 using Student’s t-test).

To determine the effect of CM shape on the expression of connection proteins typically found in the heart, we examined the proteins and RNA from net-shaped and sheet-shaped CMs. Connexin 43 (*CX43*, the primary gap junction in working CMs), plakoglobin (*GUP*) and N-cadherin (components of desmosomes and adherens junctions, respectively, located at intercalated discs in the human heart) were up-regulated (Figure 6C) in the net-shaped group. Western blots and immunostaining showed increased expression of CX43 in net-shaped compared with sheet-shaped CMs (Figure 6A, B, D, and E). The results indicated that robust contraction plays a vital role in the maturation of intercalated discs.

## DISCUSSION

In this study, we revealed that differently shaped hiPSC-CM populations from the same differentiation batch exhibit different cellular and molecular properties. Specifically, the net-shaped CMs were more mature than the sheet-shaped cells, suggesting that the physical formation of the multicellular structure could significantly affect the maturation of hiPSC-CMs. Even within the same culture, CMs exhibit various developmental or maturation statuses due to the different types of aggregates formed, which may lead to variations in gene expression profiles and contractile and ultrastructure properties. The generation of multicellular formation patterns of hiPSC-CMs is a common phenomenon, but no previous reports have described the differences among these patterns. Our study is the first to analyze the movement of net-shaped CMs and sheet-shaped CMs, and this morphological arrangement of hiPSC-CMs exerted a profound impact on their maturation status. Three typical forms of CM clusters are readily obtained using the small molecule-based differentiation method: isolated beating clusters, net-shaped CMs and sheet-shaped CMs. The isolated beating clusters were derived from a lower efficiency differentiation process. We then focused on the more reproducible net-shaped and sheet-shaped CMs, which were derived from differentiation proceeding at much higher efficiencies (80-95% TNNT2+). The percentages of ventricular and atrial cells were not significantly different in cells with the two distinct morphologies (Figure S3). Interestingly, a lower seeding density (3.0×10^3^ and 1.2×10^4^ cells/cm^2^) of hPSCs resulted in highly efficient net-shaped CM production. Different seeding densities could lead to the growth patterns of mesoderm specification and the following cardiogenesis, which eventually lead to the variation of 3D structure. This simple and effective method was used to obtain hPSC-CMs with the preferred morphologies.

The maturation of hiPSC-CMs is critical for their application in modeling of cardiac disease and in investigations of pathogenesis and regenerative medicine. Currently, several distinct approaches have been employed to promote the maturation of hiPSC-CMs, such as 3D culture methodology and the use of electrical stimulation in conjunction with stretching to mimic cardiac load, concurrently or sequentially. Other strategies include culturing in the presence of T3 thyroid hormone or insulin-like growth factor-1, the addition of laminin or native decellularized heart ECM into the hydrogel mixture and the use of stiffer substrates. Intra-, inter- and extracellular mechanical forces play vital roles in the differentiation and maturation of CMs, both *in vivo* and *in vitro* [25,26]. We recognize that mechanical stretch and electrical stimulation are widely used in 2-dimensional and 3-dimensional culture systems to improve CM maturation and function *in vitro*. However, inappropriate external stimuli may contribute to CM damage and pathological consequences [27,28]. Our results offer a new strategy to improve the maturity of hiPSC-CMs. Researchers may find that they are using CMs from different batches or even pluripotent cell lines to assess phenotypes, which are significantly affected by maturity. In the present study, the net-shaped CMs contracted so vigorously that most of them detached, with only a few cells remaining attached. The maximum motion amplitude of their wave-like movements was up to 120 μm, which was even visible to the naked eye.

We observed improved cellular myofilament structure in net-shaped CMs, with physiological hypertrophy characterized by increased cell size, a developed sarcomeric structure, sensitivity to low concentrations of Ca^2+^ channel antagonists and up-regulation of α-MHC and ANP expression, consistent with previous reports of mechanical stimulation-induced CM maturation [15,29]. Furthermore, the beating rate of the net-shaped CMs remained at 2~3 Hz, which may be another reason for enhanced CM maturation. Our real-time PCR data revealed the significant up-regulation of late cardiac-specific genes such as *CACNA1C*, *RYR2* and *KCNQ1*, *SERCA2a*, *SCN5A*, *KCNH2*, and *KCNJ2*. This finding is consistent with other studies and resembles the pacing with electrical stimulation in 3D cultures or engineered heart tissue constructs [4,11,30,31]. The net-shaped cardiac cells contracted so vigorously that much more mechanical and electrical stimulation were generated, mimicking the “exercise” effects. Moreover, the CMs feedback with building up a more mature machinery with elevated capacity.

Intercellular mechanical coupling is necessary to ensure appropriate cardiac contraction, and this is facilitated by adherens junctions and desmosomes. Adherens junctions are the anchor-point of myofibrils that enable the transmission of the contractile force from one cell to another [32,33]. In the present study, we observed that the adherens junction and the desmosomes markers in cardiac N-cadherin and plakoglobin (GUP) are up-regulated at the transcriptional level. In addition to mechanical coupling, gap junctions mediate the intercellular electrical and metabolic coupling through direct communication between neighboring cells. The up-regulation of *CX43* in net-shaped CMs also suggested enhanced hiPSC-CMs maturation.

In summary, efficiently differentiated (greater than 85%) hiPSC-CMs exhibited two distinct morphologies: sheet-like and net-like patterns of multicellular formation. The latter showed accelerated maturation, based on the sarcomeric structure and cardiac gene expression. Based on these results, the physical formation of the multicellular structures significantly affects the maturation of hiPSC-CMs. To the best of our knowledge, this study is the first to describe that variations in hiPSC-CM morphology directly result from differentiation conditions and have a profound impact on their maturation status. Using appropriate induction conditions, particularly the seeding density, the net-shaped pattern of CMs was consistently generated, providing a new strategy for improving the maturation status of hiPSC-CMs

## MATERIALS AND METHODS

### Human pluripotent stem cell culture and cardiac differentiation

AC-hiPSCs (derived from skin fibroblasts, Cellapy, Beijing, China) and NKX2.5eGFP/W-hESCs (provided by Dr. Stanley from Monash University, Australia [34]) were employed in this study. The hiPSCs were maintained on feeder-free Matrigel (Corning) in E8 medium (Stem Cell). The cells were passaged every four days at 80% confluence using 0.5 mmol/L EDTA in D-PBS without CaCl_2_ or MgCl_2_ (HyClone). The Rho kinase inhibitor Y-27632 (10 µM) was added for the first 24 hours after passaging. The cells were maintained at 37°C with 5% CO_2_.

hiPSCs were differentiated into CMs using a small-molecule-based method as previously described [34,35]. The cells were treated with 6 µM CHIR99021 (GSK-3 inhibitor, Selleck Chemical) in basal medium (RPMI supplemented with B27 without insulin (both from Invitrogen, Carlsbad, CA, USA)) for 48 hours (day 0 to day 2). The next day, the medium was changed to basal medium. On day 4, the medium was changed to basal medium with the inhibitor Wnt protein 2 (IWP-2) (Selleck Chemical) for 2 days. The cells were cultured in the maintenance medium (RPMI supplemented with regular B27 (Invitrogen)) starting from day 7, with media changes every 1-2 days. Compared with FBS, the RPMI and B27 culture condition has the advantage of preventing fibroblast overgrowth. Regular media changes were performed to limit pH alterations.

CMs were dissociated via treatment with the commercially available CardioEasy CM dissociation enzyme set (Cellapy, Beijing China). Briefly, the collagen-based enzyme 1 was applied for 30 min at 37°C, the trypsin-based enzyme 2 was applied for another 5 min, and then CMs were pipetted up and down to dislodge the cells and disrupt the aggregates. The cells were transferred to a 15-ml conical tube, the remaining volume was filled with RPMI/B27 with insulin, and the sample was centrifuged for 5 min at 200×g at room temperature.

### Immunostaining and imaging analyses

Mitochondria in live cells were labeled with MitoTracker Red (Life Technologies) [24,36] at a concentration of 50 nM for 10 min at 37°C. Cells were washed once with warm PBS and new warm culture medium was added immediately before imaging. Nuclei were visualized by incubating live cells in medium containing 1 µg/mL Hoechst 3342 (Life Technologies) for 5 min at 37°C, followed by the addition of fresh medium.

The cells were fixed with 4% paraformaldehyde, permeabilized in PBS containing 0.2% Triton X-100 and blocked with 3% BSA. Samples were stained with the following primary antibodies: rabbit polyclonal anti-cardiac troponin I (cTnI) (1:100; Santa Cruz Biotechnology), mouse monoclonal anti-cardiac troponin T (cTnT) (1:100; Santa Cruz Biotechnology), rabbit polyclonal anti-cardiac MYL2 (1:100, Novus Biologicals), mouse monoclonal anti-MYL7 (1:100, Santa Cruz Biotechnology), rabbit polyclonal anti-α-actinin and rabbit polyclonal anti-connexin-43 (1:100, Santa Cruz Biotechnology). After three washes with PBS, the slides were stained for 1 hour with the following secondary antibodies: goat anti-mouse IgG Alexa Fluor488, goat anti-rabbit IgG Alexa Fluor594, goat anti-mouse IgG Alexa Fluor594, and goat anti-rabbit IgG Alexa Fluor488 (Invitrogen, these antibodies were used at dilutions of 1:500-1000). All slides were counterstained with 4,6-diamidino-2-phenylindole (DAPI, 300 nM, Invitrogen) for 5 min. Fluorescence images were obtained with an Olympus IX81 inverted microscope using a Hamamatsu C4742-95 camera.

Bright-field images were acquired using Toupcam mounted to an Olympus CX41 microscope. Images were quantified using ImageJ software and standard analysis plugins. The cell area, cell perimeter, and cell circularity index of each cell was analyzed. The circularity index was calculated as 4πA/P^2^, where A is the area, and P is the perimeter.

### Flow cytometry

hiPSC-CMs were collected on day 15 of differentiation, dissociated using CardioEasy CM dissociation enzyme (Cellapy), filtered through a 40-µm cell strainer (Falcon) and fixed with fixation buffer (BD Biosciences) for 15 min at room temperature. Fixed cells were washed with Perm/Wash buffer (BD Biosciences) and then analyzed for eGFP expression by FACS analysis or incubated with a 1:100 dilution of a mouse monoclonal anti-cardiac troponin T (cTnT) antibody for 30 minutes at room temperature. Cells were then incubated with a 1:100 dilution of Alexa Fluor 488-conjugated anti-mouse secondary antibodies (Life Technologies) for 30 min. The cells were then washed and were analyzed using FACS analysis. The analysis was performed with the FlowJo program.

### RNA extraction, whole-transcriptome sequencing and quantitative real-time PCR

Total RNA was extracted with TRIzol (Invitrogen) according to the manufacturer’s protocol, followed by DNase I treatment to eliminate DNA contamination. The mRNA was enriched and cleaved into short fragments. The double-stranded cDNA was synthesized and purified before sequencing adaptors were ligated to the fragments. For quality control, an Agilent 2100 Bioanalyzer and the ABI StepOnePlus Real-Time PCR System were used to qualify and quantify the sample library. The library products were finally sequenced using an Illumina HiSeq 2000. Clean reads of at least 11 M were obtained from each sample.

For quantitative real-time PCR, 1 μg of total RNA was reverse transcribed into cDNAs using the GoScript reverse transcription system (Promega). The gene expression levels were analyzed by quantitative reverse transcriptase PCR (qRT-PCR) performed with 2 × SYBR Master Mix (Takara, Otsu, Shiga, Japan) using an iCycler iQ5 (Bio-Rad, Hercules, CA, USA). The relative quantification was calculated according to the △C_T_ method. Table S1 shows the primers used.

### Transmission electron microscopy

The cells were fixed with 4% formaldehyde and 1% glutaraldehyde in 0.1 M Sorenson’s buffer (pH 7.2) for 1 hour. The cells were then post-fixed with 1% OsO_4_ in Sorenson’s buffer for 1 hour. After dehydration, the cells were embedded in Beam capsules and baked in an oven at 60°C for 48 hours. Thin sections (60 nm) were cut on an MT-7000 ultramicrotome, stained with uranyl acetate and lead citrate, and examined on a JEM-2100 electron microscope. Sarcomere thickness was measured as the length of a single Z-line.

### Drug response

Single contracting iPSC-CMs were treated with pharmaceutical agents for 10 min for immediate analysis followed by washout. The respective concentrations of each drug tested are listed in Table S2.

### Video analysis

The cyclic motion of the tissue was analyzed on the video acquired from the Olympus CX41. The behavior of the tissue motion was quantitatively determined from the post-processing of the acquired video. The amplitude of the cyclic motion was measured on the M-mode image, which represented the temporal change of a certain line in the video frame. A cross-correlation-based speckle tracking method that is widely used in the field of ultrasound elastography [37] was applied to the video data to estimate the tissue velocity in the present study. The local velocities of the tissue as a function of time were calculated, and the maximum velocities for each cycle and the motion period were determined.

### Data analysis and statistics

All data are expressed as means±standard errors of the means (S.E.M.). Mapping data were normalized to a 0-1 scale, X/$\overset{-}{x}$_sheet-d15._ Statistical significance was evaluated using Student’s t-test for 2 groups or one-way ANOVA for comparisons of multiple groups. Differences with values of P<0.05 were considered statistically significant.

## Supplementary Material

Supplementary File

Supplemetary Movie S1

Supplemetary Movie S2
