# Supplementary material for: A net-shaped multicellular formation facilitates the maturation of hPSC-derived cardiomyocytes through mechanical and electrophysiological stimuli
Source: Aging (Albany NY). 2018 Apr 14;10(4):532–48. doi: 10.18632/aging.101411 (PMC5940117; doi:10.18632/aging.101411)
Supplement: Supplementary File [file aging-10-101411-s001.pdf]

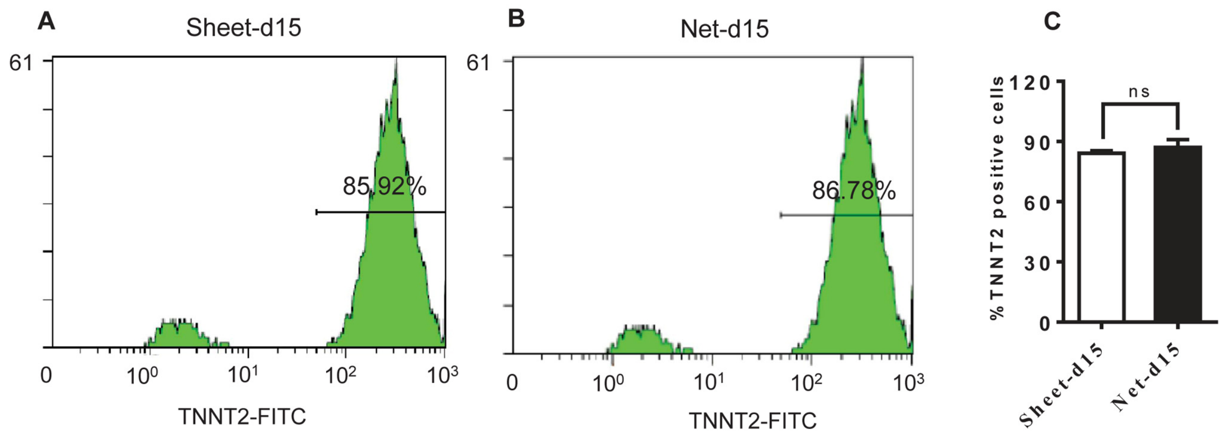

Supplementary Figure S1. Percentage of TNNT2+ cells in two different growth patterns measured by flow cytometry on day15 of differentiation. (**A**, **B**) Representative flow-cytometry of Sheet-d15 (**A**) and Net-d15 (**B**). (**C**) Quantification of TNNT2+ in sheet-d15 and net-d15. All data were normalized to the sheet-day 15 group and are expressed as the means ±S.E.M. * Statistically significant differences between individual groups (n≥3; *P<0.05, ns, no significant).

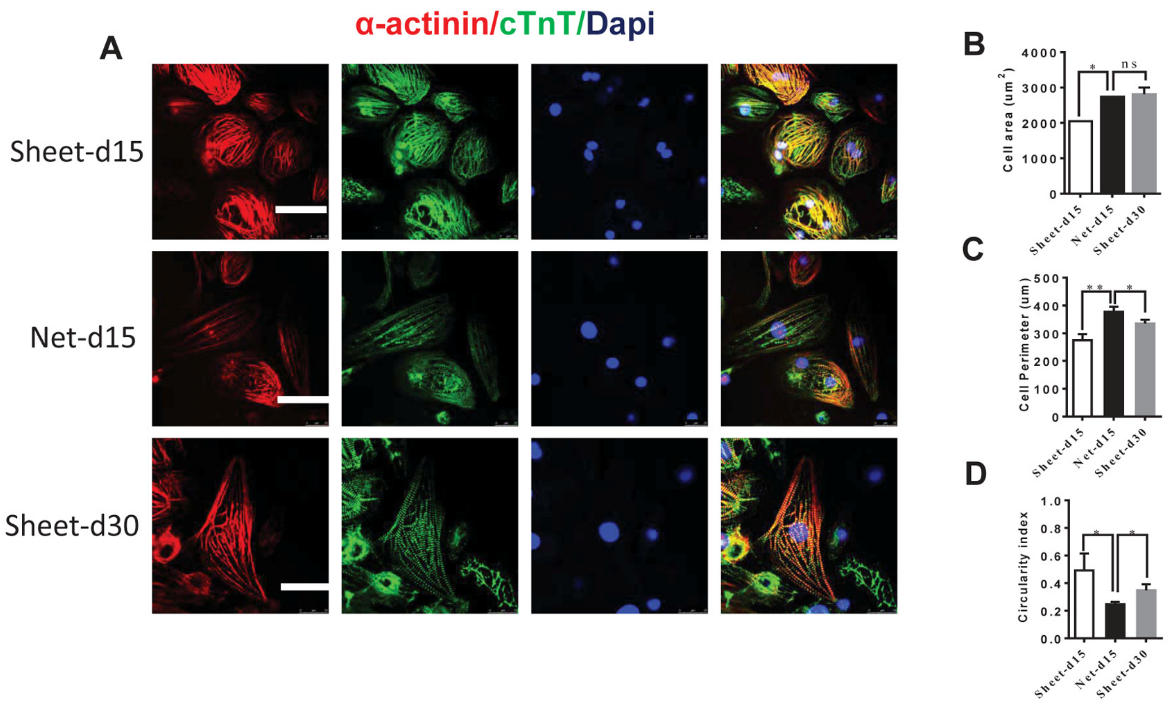

Supplementary Figure S2. The Morphology of sheet-d15, net-d15 and long-term cultured CMs(Sheet-d30). (**A**) Immunostaining of different shaped hiPSC-CMs 15 days and long-term cultured CMs after dissociated single cells. a-actinin(red) cTnT (green), DAPI (blue). Up panel: sheet-d15 Midle panel: net-d15; Down panel: Sheet-d30. (**B, C, D)** Cell area, cell perimeter, circularity index (n=51 in net-d15, n=45 in sheet-d15 and n=66 in sheet-d30). All data were expressed as the means ± S.E.M. * Statistically significant differences between individual groups (n≥3; *P<0.05, **P<0.01, ***P<0.001. Scale bar: 50 µm.

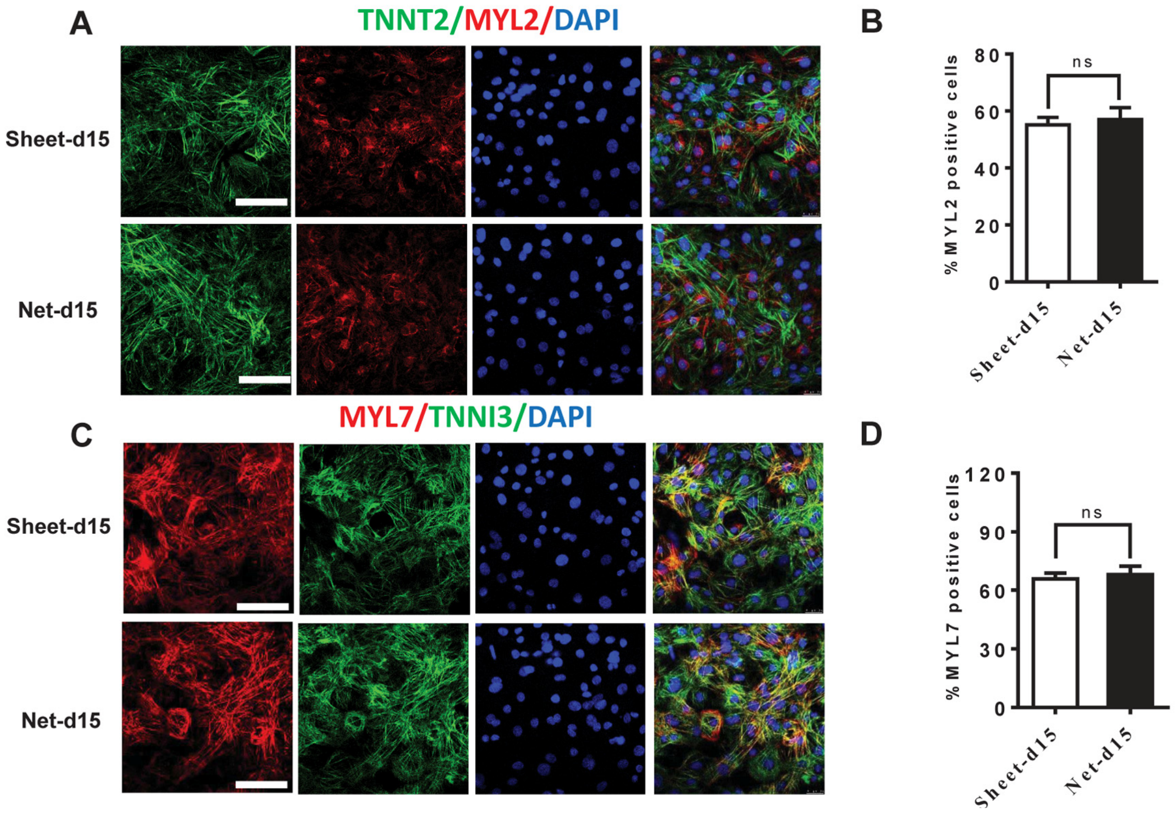

Supplementary Figure S3. Characterization of cardiomyocytes subtypes. (**A**,**C**) Immunofluorescence staining of two shapes of hiPSC-CMs with antibodies to the indicated proteins. (**A**) TNNT2(Green), MYL2(Red); (**B**) TNNI3(Green), MYL7(Red); (**B**,**D**) The percent of MYL2 (**B**) and MYL7 (**D**) positive cells. All data were normalized to the sheet-day 15 group and are expressed as the means ± S.E.M. *Statistically significant differences between individual groups (n≥3; *P<0.05, **P<0.01, ***P<0.001. Scale bar: 50 µm.

Table S1. Primer sequences used for real-time quantitative polymerase chain reaction analysis

Gene	Forward 5'-3'	Reverse 3'-5'
MYL2	TACGTTCGGGAAATGCTGAC	TTCTCCGTGGGTGATGATG
LRRC39	CTGGGTACTCTTGTTCTCAG	TCCCGTTCCTCTTCTTCATC
TNNI3	CTGCAGATTGCAAAGCAAGA	CCTCCTTCTTCACCTGCTTG
GAPDH	GTGGACCTGACCTGCCGTCT	GGAGGAGTGGGTGTCGCTGT
KCNH2	AATCGCCTTCTACCGGAAAG	CACCATGTCCTTCTCCATCAC
KCNQ1	TCTGTCTTTGCCATCTCCTTC	CCTCCATGCGGTCTGAATG
KCNJ2	AAGACGGTATGAAGTTGGCC	CGGGTGTGGACTTTACTCTTC
SCN5A	CTGACCTCACCATCACTATGTG	GCTGTGAAAATCCCTGTGAAG
CACNA1C	TTCGTCATCGTCACCTTTCAG	TGTACTGGTGCTGGTTCTTG
SERCA2A	GATCACACCGCTGAATCTG	AGTATTGCGGGTTGTTCCAG
TNNT2	AGCATCTATAACTTGGAGGCAGAG	TGGAGACTTTCTGGTTATCGTTG
MYH6	TCTCCGACAACGCCTATCAGTAC	GTCACCTATGGCTGCAATGCT
MYH7	GGCAAGACAGTGACCGTGAAG	CGTAGCGATCCTTGAGGTTGTA
MYL7	GAGGAGAATGGCCAGCAGGAA	GCGAACATCTGCTCCACCTCA
GJA1	CAATCACTTGGCGTGACTTC	AAAGGCAGACTGCTCATCTC
CACNA1C	CAGAGGCTACGATTTGAGGA	GCTTCACAAAGAGGTCGTGT
RYR2	AGAACTTACACACGCGACCTG	CATCTCTAACCGGACCATACTGC

TNNT2*,** cardiac troponin-T; ***LRRC39*,** leucine-rich repeat-containing protein 39, ***MYH6, myosin heavy chain 6; ***MYH7***, myosin heavy chain 7; ***MYL2***, myosin regulatory light chain2; ***MYL7***, myosin regulatory light chain 7;***SERCA2***, sarco/endoplasmic reticulum Ca^{2+} ATPase isoform 2; ***RYR2***, ryanodine receptor 2; ***SCN5A***, NaV1.5 voltage-gated Na+ channel ; ***CACNA1C***, L-type voltage-gated Ca2+ channels; ***KCNH2***, the human *Ether-à-go-go-*Related Gene; ***KCNQ1***, Kv7.1; ***KCNJ2***, Kir2.1 inward-rectifier potassium ion channel

Table S2. Drugs informations for using to assess the pharmacological responses

Drug	Target	Brand	Cat.No	Concentrations Tested
Isoproterenol	β-adrenergic receptor agonist	sigma	I5627-5G	0,10,100,1000nM
Verapamil	CaV1.2 channel antagonist	sigma	V4629-1G	0,10,100nM
Nifedipine	L-type Ca2+ channel antagonist	sigma	N7634-5G	0,10,100nM
Nisoldipine		sigma	N0165-10MG	0,10,100nM

SUPPLEMENTARY MOVIES

Please browse the Full Text version to see :
Movie S1. Move motions of Net-day 15 CMs. Move motions of Net-day15 CMs.

Movie S2. Move motions of Net-day 15 CMs. Move motions of Sheet-day15 CMs.
